# The Need for Restructuring the Disordered Science of Amorphous Drug Formulations

**DOI:** 10.1007/s11095-017-2174-7

**Published:** 2017-05-18

**Authors:** Khadijah Edueng, Denny Mahlin, Christel A. S. Bergström

**Affiliations:** 1 Department of Pharmacy, Uppsala University, Uppsala Biomedical Centre, P.O. Box 580, SE-75123 Uppsala, Sweden; 2 Kulliyyah of Pharmacy,, International Islamic University Malaysia, Jalan Istana, 25200 Bandar Indera Mahkota, Pahang Malaysia

**Keywords:** amorphous solid dispersion, computational tools, crystallization, dissolution, stability

## Abstract

The alarming numbers of poorly soluble discovery compounds have centered the efforts towards finding strategies to improve the solubility. One of the attractive approaches to enhance solubility is via amorphization despite the stability issue associated with it. Although the number of amorphous-based research reports has increased tremendously after year 2000, little is known on the current research practice in designing amorphous formulation and how it has changed after the concept of solid dispersion was first introduced decades ago. In this review we try to answer the following questions: *What* model compounds and excipients have been used in amorphous-based research? *How* were these two components selected and prepared? *What* methods have been used to assess the performance of amorphous formulation? *What* methodology have evolved and/or been standardized since amorphous-based formulation was first introduced and to *what* extent have we embraced on new methods? Is the extent of research mirrored in the number of marketed amorphous drug products? We have summarized the history and evolution of amorphous formulation and discuss the current status of amorphous formulation-related research practice. We also explore the potential uses of old experimental methods and how they can be used in tandem with computational tools in designing amorphous formulation more efficiently than the traditional trial-and-error approach.

## Background

Looking back in the scientific literature gives an interesting historic view on the use of amorphous solid dispersion (ASD) as a means of improving the dissolution of poorly soluble drugs. A number of publications from the 1960s reported the use of co-precipitates as formulation for poorly soluble drugs. Predominantly, these systems included a drug and a soluble component in an intimate mixture formed by co-precipitation from a common solvent via solvent evaporation. The water-soluble component was typically a polymer, for example polyvinylpyrrolidone (PVP) (1–3), or a low molecular weight compound such as urea (4).

In the 1960s, the primary aim of solid dispersions was to achieve improved dissolution for a poorly soluble drug. As it became apparent that ASD often results in supersaturation upon dissolution, the aim nowadays is predominantly to attain supersaturation. In 1960, Mullins and Macek (5) published a pioneer study of the dissolution behavior of a neat amorphous compound, novobiocin. In the study, the amorphous form and a salt of the drug were used and compared with the crystalline form. The study included evaluation of bioavailability. The authors screened 23 potential nucleation inhibitors which were added to reduce precipitation by crystallization of the drug from the supersaturated solutions formed during dissolution of the amorphous novobiocin. A few of these were identified as reducing precipitation during dissolution, one of these being PVP which is still used for the same purpose. Another early publication worth mentioning is that by Simonelli *et al*. (1), in which a theoretical model for dissolution of a solid dispersion of sulphathiazole and PVP was proposed.

The concept of a “solid dispersion” quickly evolved after 1961, when Sekiguchi and Obi (6) showed how to produce dispersion of a poorly soluble drug in a water-soluble carrier by forming an eutectic mixture for the purpose of improving oral absorption. Goldberg *et al*. (4) reviewed a number of reports on improving dissolution by forming eutectic mixtures but, importantly, the superior effects of a ‘solid solution’ were emphasized. The article also provided a theoretical description of the behavior of the crystalline phase in these solid mixtures. Solid dispersions and solid solutions that included an amorphous phase were reviewed later by Chiou and Riegelman (7). It was two years earlier (in 1969) (8) that the authors had introduced the term “glass solution” as a potential drug formulation concept in a study on the co-melting and solidification behavior of griseofulvin with citric acid. However, the term “glass solution” was replaced by the broader term “solid dispersion” and was then absent from the pharmaceutical literature until it was revived by Forster, Hempenstall and Rades in a series of publications in the early 2000s (9–11). It is interesting to note that, since 2009, the same type of system (drug and water-soluble, low-molecular-weight compound in an amorphous state) has often been referred to as “co-amorphous” (12–14). This type of formulation has attracted a great deal of interest for use with poorly soluble compounds.

In 1987, Doherty and York used the term ASD to describe the amorphous mixture of furosemide and PVP (15). The interactions between the components were studied using infrared spectroscopy and nuclear magnetic resonance (NMR) techniques, and the idea of strong interactions between the two components being important for amorphous formulation performance was introduced (15). In the same year, El-Hinnawi and Hajib used the same techniques for studying amorphous ibuprofen-PVP (16). Both these publications indicated that the systems studied were actually “glass solutions”, but in the publications, the authors used the general term “solid dispersion”. After the publication of the highly cited article by Taylor and Zografi on the interactions between indomethacin and PVP in 1997 (17) ASD became a recognized terminology and is widely used thereafter in pharmaceutical research for this type of amorphous mixture.

In the 1990s, a number of publications by Zografi and co-workers showed how the glass transition temperature (T_g_) is dependent on the composition of the amorphous mixture (18,19) and the role of water sorption was also evaluated (20,21). This was the starting point for numerous investigations into the role of T_g_ and molecular mobility in the physical stability of amorphous systems, and their relationship with the interactions between polymeric stabilizers and the amorphous component. In 1995, Hancock *et al*. (22) proposed a relationship between T_g_ and the storage stability, stating that if an amorphous system was stored 50°C below its T_g_, the shelf life would be prolonged for years. By the turn of the century, a range of research groups were interested in molecular mobility, as exemplified by the number of reports of different techniques for measuring mobility, such as dielectric spectroscopy (19,23–25), relaxation by differential scanning calorimetry (DSC) (22,26) and NMR (27).

It is interesting to observe in the research reports from the 1990s that the polymeric additive used to reduce molecular mobility in amorphous drugs was often seen as a stabilizing additive for the amorphous phase, rather than as a water-soluble matrix as in the solid dispersions in the previous works. The notation “solid dispersion” remained, however, and this terminology now includes most types of amorphous drug/polymer combinations.

Although the solubility advantage of amorphous compounds has been of great interest for a long time, the problem of unstable supersaturation causing difficulties with measuring the amorphous solubility has impeded efforts to better understand these systems. This was emphasized by Hancock and Parks in 2000 (28), who reported only five published amorphous solubility studies, including the one on novobiocin from 1960. After 2000, the number of publications on ASDs has increased tremendously. In this review the most recent of these have been studied to analyze the general understanding of these systems. In focus of the analysis are the drug properties, excipient selection, choice of production method and exploration of performance during dissolution, to provide an updated view on the gaps currently present in this research field.

## Current Status of Research on Amorphous Formulations

A literature review was carried out to provide an updated view of compounds, excipients and methods currently being explored for the production of stable amorphous drug formulations. One hundred-one articles relevant to the topic of interest published between January 2011 and December 2016 were found using the PubMed database and the search string “amorphous AND solid AND dispersions AND poorly AND soluble AND drug AND polymers OR polymer”. The results were sub-classified into four important aspects of amorphous formulation and assessment: (i) which compounds were being investigated during this time frame?; (ii) how were excipients selected?; (iii) what was the processing technology or method of preparation?; and (iv) how was the performance of the amorphous formulation assessed (physical stability, apparent solubility and dissolution profiles)?

### Selection of Model Compound

The current trend for newly discovered compounds to be poorly soluble (70–90% of all new compounds are poorly soluble (29)) has been attributed to the chemical processes used for synthesis (30), the organizational behavior of large research organizations (31,32), and the target biology (33–35). The molecular features that result in limited solubility and/or a reduced dissolution rate have been defined. Typically these features are used to divide drug molecules into two major categories: molecules showing solid-state-limited solubility (also colloquially called ‘brick-dust’ molecules to show that they form strong, tightly associated intermolecular bonds in a dense crystal lattice) and molecules showing solvation-limited solubility (also colloquially called ‘grease-ball’ molecules to show that they have poor interactions with water molecules and tend to aggregate when exposed to water). Compounds with high (>200°C) melting points (T_m_) and moderate lipophilicity (a partition coefficient between octanol and water, logP, of <2) are classified as ‘brick-dust’ molecules, whereas those with high lipophilicity (logP >3), higher molecular weight (~MW >400 g/mol), and a poorly integrated electron system are typically identified as ‘grease-ball’ molecules (36,37). Some compounds have both a high T_m_ and a high logP which indicate limited solubility from both the solid state and solvation aspects.

Amorphization is an attractive approach to increase both the solubility (where the solubility of the amorphous form rather than the crystalline form is the important factor) and the dissolution rate (38–43). This approach should be possible for both categories of drug discussed above, but brick-dust compounds will probably benefit more from amorphization. This is because, in order to increase the apparent solubility and the dissolution rate of the solid state-limited compounds, the strong crystal lattice has to be weakened. This can be achieved by using another polymorph (44), by introduction of co-formers to produce co-crystals (45,46), by selecting a salt of the drug (47), by making use of prodrug strategies (48), nanocrystal technologies (49,50) or by using amorphization (or formation of amorphous nanoparticles) (51–53). For a long period, amorphization was not the first choice because of the inherent instability issues resulting from the high energy form of the amorphous solid material (as indicated in the Background section). However, since poor solubility is now a hurdle for so many newly discovered compounds, all means possible are currently investigated in order to improve the compound properties (54). It appears that the scientists formulating new compounds have embraced the amorphous solid dosage forms to a greater extent during the last five years than was apparent during the early 2000s. This was to some extent confirmed by a search of the PubMed database using the search string described above. Twenty-seven articles on amorphous solid dispersions were published between January 2000 and December 2010 (eleven years), which is only 27% of the number reported for the last six years (the period covered in this review).

In an amorphous solid, the strong intermolecular bonds within the crystal lattice are lost and there is no long-range order; this more random order in the interactions between the drug molecules results in an increase in the free energy of the amorphous system. However, improvement in the overall dissolution profile of the amorphized compound is not only dependent on disruption of the crystal lattice. When producing, for example, an ASD, the solubility and dissolution are also often improved by the reduction in particle size and the increased hydrophilicity (and resultant improved wettability) caused by the addition of hydrophilic and/or amphiphilic excipients (51). Thus, the formation of ASDs can be beneficial for both brick-dust and grease-ball drug molecules.

Compounds which demonstrate good glass-forming ability (GFA) and have a low crystallization tendency (good physical stability) are potential candidates for formulation as ASD (55–59). In 2010, Baird *et al*. classified 51 organic compounds into three groups based on their GFAs and crystallization tendencies (56). The compounds were classified as glass formers (classes II and III) or non-glass formers (class I), according to their crystallization behavior during the heating/cooling/heating cycle, as measured by differential scanning calorimetry (56). Class I compounds were defined as those that crystallized during the period as the melt cooled, thus not existing in an amorphous form at, for example, room temperature. Class III compounds were defined as those forming a stable glass on melting that did not recrystallize during either cooling or the second heating. Class II compounds were defined as non-stable glass formers that recrystallized during the second heating. A similar but larger study was performed by Alhalaweh *et al*. in 2014. They used computational modeling to relate the physicochemical properties of 131 compounds to their GFAs and crystallization tendencies (60). In a different but related study, Wyttenbach *et al*. studied 54 compounds to ascertain the extent to which the Prigogine-Defay ratio could be used to predict the GFA (61). The physical stability (dry state) of amorphized compounds has also been explored (61) and Nurzyn’ska *et al*. recently presented data on the parameters related to the long-term stability of amorphous drugs (62). The findings from recent studies on GFA and crystallization behavior can be summarized thus:

- Characteristics indicating glass-forming compounds include: MW >300 g/mol, torsional bonds (τ) >4, and effective hydrogen bond number (HBN_eff_) < 6 x 10^−3^ (60,61,63).
- Molecules with more branching, less symmetry, and a low number of benzene rings are good glass formers (64).
- Compounds with a high crystallization temperature (>100°C), a high Hückel pi atomic charge for carbon atoms, and the capacity to form hydrogen bonds appear to be Class III glass formers (60).
- A high crystallization temperature (>100°C), a high Hückel pi atomic charge for carbon atoms, and a high aromaticity are associated with physically stable glass at room temperature (63,65).

Improved understanding of the physicochemical properties influencing the GFA and crystallization tendency of amorphous compounds is required to establish a science-based decision gateway on when to use an amorphous formulation to improve the solubility of poorly soluble compounds.

**Fig. 1:**
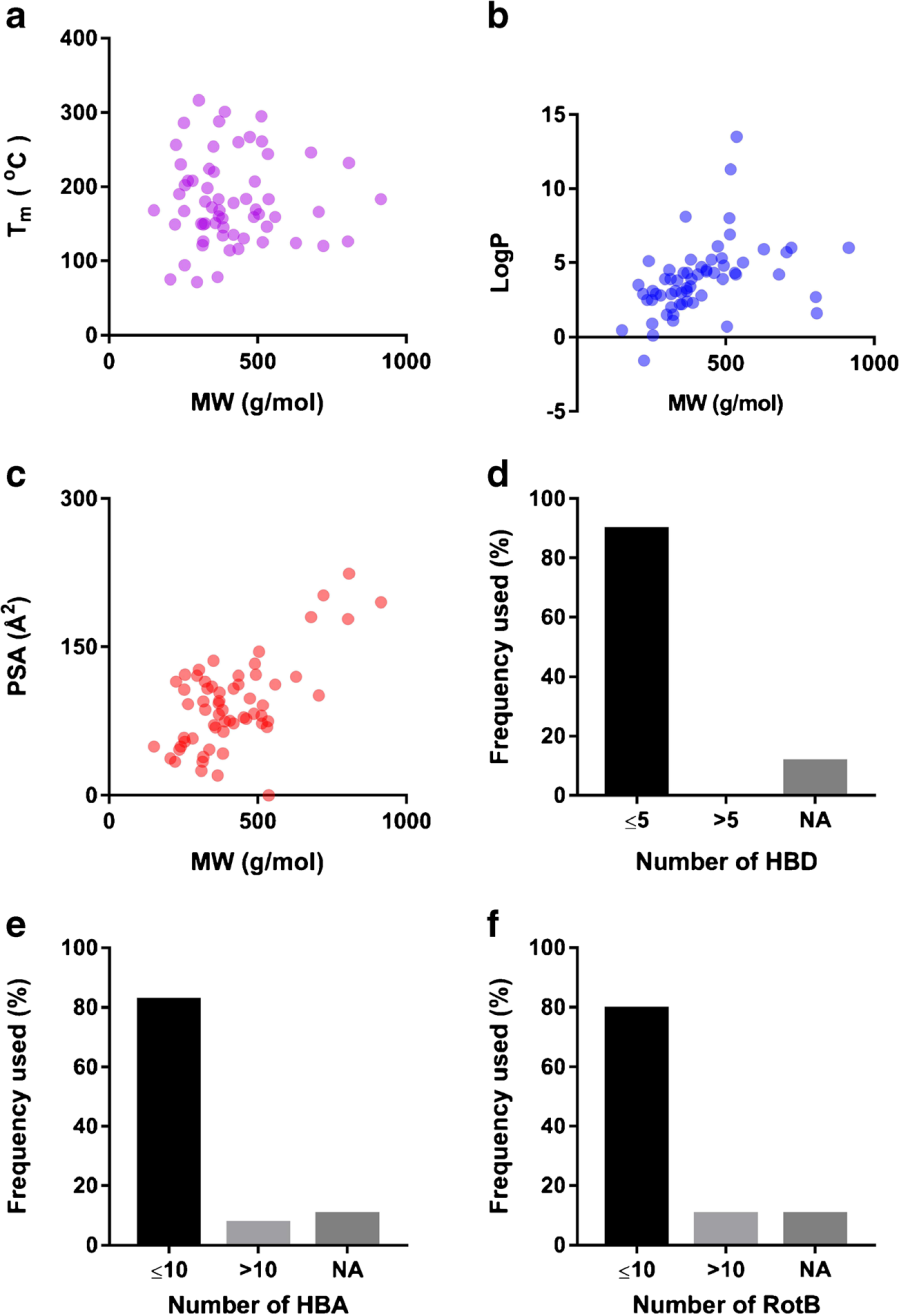
Distribution of the physicochemical properties of model compounds: molecular weight (MW), melting point (T_m_), partition coefficient between octanol and water (LogP), polar surface area (PSA), number of hydrogen bond donors (HBD), number of hydrogen bond acceptors (HBA), and number of rotatable bonds (RotB).

Consequently, we reviewed the recent literatures for commonly studied compounds formulated as amorphous systems to extract information about the physicochemical space currently being explored for this formulation area (Fig. 1). In particular, we investigated the relationships between amorphization and molecular size and complexity (MW, number of heavy atoms, and molecular complexity), T_m_, logP, polar surface area (PSA), number of hydrogen bond donors and acceptors (HBD and HBA), and number of rotatable bonds (RotB). These physicochemical properties were analyzed from two different perspectives: (i) Lipinski’s rule-of-five (Ro5) and (ii) molecular properties described above (brick-dust *vs*. grease-ball, GFA, and crystallization tendency). In short, according to Lipinski Ro5, poor intestinal permeation and/or poor solubility (and hence absorption) is expected from a compound with MW >500 g/mol, logP >5, HBD >5, and HBA >10 (66). We also included T_m_ since a high T_m_ has previously been negatively associated with GFA (55,56).

Sixty-eight model compounds reported in 101 articles were analyzed with respect to chemical space. Most of the model compounds studied for amorphous formulation fell within the Ro5 definition (Fig.1). The increasing number of lead compounds falling outside the Ro5 chemical space (30) is clearly not mirrored in the scientific publications within this field. Among the articles reviewed, only 31% of the compounds were regarded as possibly poorly soluble by violating at least one of the Lipinski criteria. The Ro5 provides relatively general characteristics for compounds to predict good or poor absorption without specifying which formulation strategy is the best for any particular compound. For instance, the optimal formulation strategy will differ according to whether the compound has limited solubility or limited intestinal permeation as well as whether the compound is solid state-limited (brick-dust) or solvation-limited (grease-ball) in their solubility. An early understanding of which of these limitations is in play is therefore warranted. That allows appropriate, scientifically sound judgements to be made on which formulation strategies (i.e. which excipients, dosage forms, methods of preparation, etc.) should be used for a particular compound with known molecular properties and limitations. Subsequently, the performance (i.e. physical and chemical stability, dissolution, and intestinal permeation) of the selected formulation could also be properly understood (30,36,37,56,60,63,65,67–69).

Although amorphization as a formulation strategy could work for any group of compounds, the success of the formulation will nevertheless still depend upon inherent factors such as the physicochemical properties of the compounds. Fig. 1 shows that the compounds that were selected for amorphous formulation studies mainly fell within Lipinski Ro5 drug-like space; in that regard they were not model compounds of particularly problematic compounds. On the other hand, from the perspective of GFA and crystallization tendency (and thus stability), most of the studied compounds included in this review had properties that indicated good GFA, including high MW (>300 g/mol), a flexible molecular structure (indicated by a high number of RotBs), a high number of heavy atoms, and a reasonable number of HBDs. A high number of HBAs, a high PSA, and a high logP appear to have a positive but small influence on amorphous stability according to the study by Nurzyn’ska and co-workers in 2015 (62). Although several studies have indicated similar molecular properties as being desirable for producing stable amorphous compounds (56,60,62,63,65), applying strict cut-off values to differentiate between desirable and undesirable properties when predicting whether a poorly soluble compound would be a potential candidate for amorphous formulation should be made with caution. For example, experimental methods for determining GFA or crystallization tendency differ significantly, which could result in slightly different values. Furthermore, as shown in Fig.1, the relationships and interplay between the different physicochemical properties of the compounds are not always proportional, and hence they should be considered collectively rather than individually through the use of, for example, multivariate data analysis (61,64,65).

### Selection of Excipient

According to the International Pharmaceutical Excipient Council, pharmaceutical excipients can broadly be regarded as any pharmacologically inert component of a pharmaceutical formulation that is not the active pharmaceutical ingredient (API) and that is added intentionally to the formulation. The excipients in pharmaceutical formulations can serve several purposes, including those related to processing, esthetic enhancement, optimization of product performance, and improvements in patient compliance (70).

Excipients are often required in amorphous formulations as amorphous compounds tend to be unstable on their own. The high free energy in the amorphous system is the driving force for the transformation of the compound to its more stable (low energy) crystalline form. However, there are currently no established recommendations on how to select the proper excipients for the formulation. Excipients are added at an early stage in the formulation design to inhibit or delay crystallization of the amorphous compound, which subsequently influences the physical stability, dissolution rate, extent of supersaturation and ultimately the bioavailability of the drug *in vivo*. Analysis of the articles included in this review revealed that several factors influence the choice of excipients for amorphous formulations; these were not only closely related to the relevant amorphous formulation per se, but were also related to the goals of the studies performed.

Of the 101 articles reviewed, the motivation behind the addition of excipients was stated in 64%, while the authors did not specify the reason(s) for selecting a certain excipient in the remaining articles (71–99). Some of the reasons given for excipient selection included reliance on previous reports of the use of similar excipients and their historical applicability in ASDs and oral dosage forms (100–125); the physicochemical properties and functions of the excipient, possible interactions between the compound and the excipient, and the miscibility/immiscibility of the compound and excipient (105–108,111,112,114,126–148); the processability or suitability of the excipient for the preparation method (73,105,119,127,149–152); and the possibility of designing a controlled-release profile and/or pH-dependent release of the compound from the amorphous formulation (99,100,107,123,153–156). An attempt to tailor and customize excipients by modifying the functional groups which could be potentially used in ASD formulations has also been reported (157). In some other studies, more unconventional excipients were explored to investigate their potential use in oral pharmaceutical formulations, with limited reports on their inclusion in amorphous dosage forms (113,158–160). The excipients used in these cases were added for many different reasons, not only to provide amorphous stability (i.e. in the solid state and solution).

Despite the lack of any established and standard recommendations on excipient selections, several studies reported the use of theoretical and calculated mixing, miscibility and solubility parameters to determine drug–excipient miscibility and interaction. These are particularly used as a means to estimate the homogeneity of the mixture at a particular ratio, which is postulated to influence the stability of the resulted amorphous system. Among others, these include the calculation of the Hansen solubility parameters (104,126,146,154,161) and the Flory-Huggins theory (94,150).

**Fig. 2:**
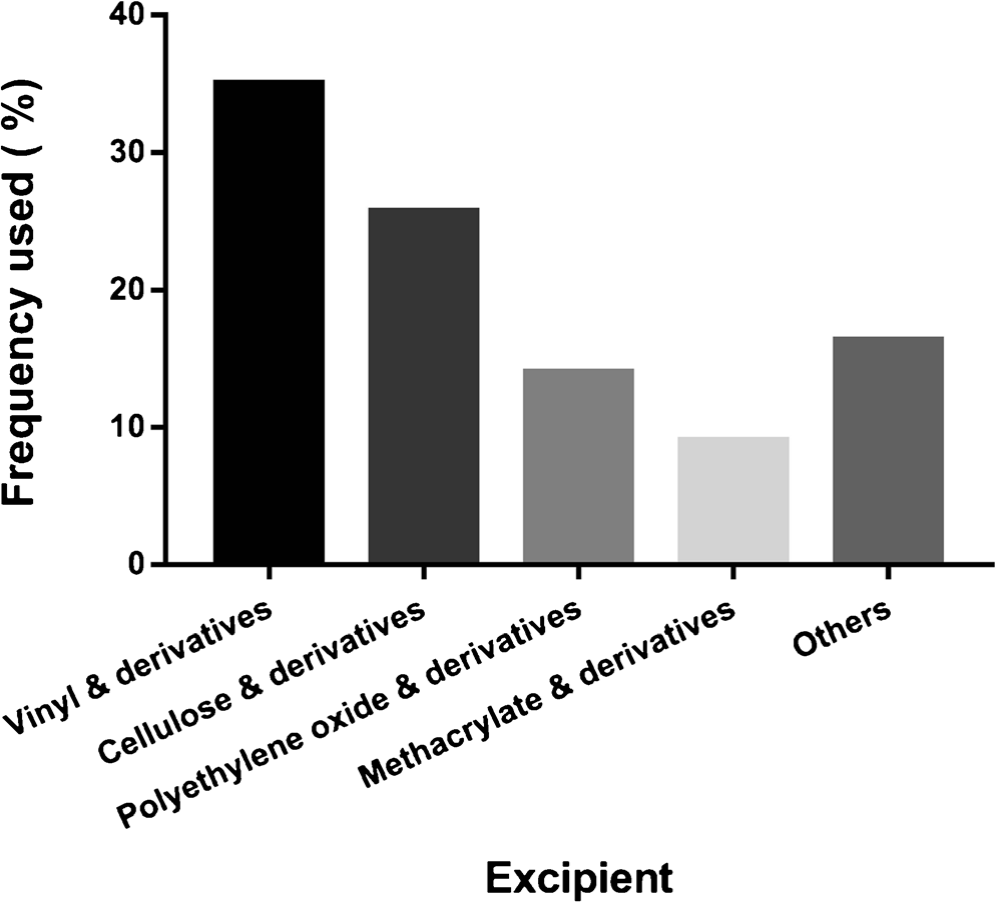
Excipient groups included in amorphous formulations.

The literature review also revealed that polymers were by far the most commonly used excipients for amorphous formulations, comprising 84% of all the reported excipients. The excipients reported in the reviewed articles are summarized in Fig.2. The polymer excipients were categorized into four groups: vinyl, cellulose, polyethylene oxide, and methacrylate and their derivatives. Vinyl and cellulose polymers were the most commonly used (35% and 26%, respectively), followed by polyethylene oxide (14%) and methacrylate (9%) polymers. The remaining 16% of the excipients used in amorphous formulations included surfactants, sugars, and alginates and derivatives thereof. These excipients were used either alone or in combination. When combined, the excipients were taken from different groups and normally served different functions aimed at improving the overall properties of the amorphous formulation. Generally, the amount of excipient and the excipient-to-compound ratio were based on the miscibility of the excipient with the compound and the ratio that would provide the best dissolution profile and/or stability performance (71,99,125–127,144,159,162).

### Selection of Preparation Method

**Fig. 3:**
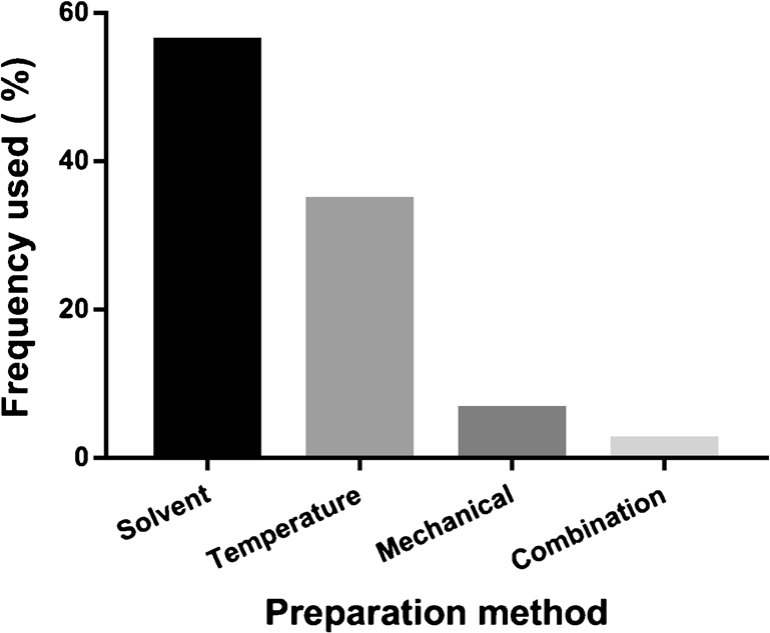
Methods of preparing amorphous formulations.

Amorphization can be achieved via several different preparation techniques. These techniques can be classified as solvent-based, temperature-based (fusion) and mechanical-based (activation); sometimes these methods are used in combination. Solvent-based methods include spray-drying, freeze-drying, precipitation, solvent evaporation, use of a confined impinging jet reactor, supercritical fluid methods, and different types of electro-spraying; temperature-based methods include the classical melt-quenching/quench-cooling methods and hot-melt extrusions. Various milling techniques were herein categorized as mechanical-based. Some preparation methods that have been combined to achieve amorphization include hot-melt extrusion and electrospinning and solvent-antisolvent precipitation and sonication (sonoprecipitation). Fifty-six percent of the reviewed methods used in preparing amorphous formulations were solvent-based, while 35% were temperature-based. Only 7% were mechanical-based and 2% used a combination of two methods (Fig. 3). Most of the methods reported in this review were used at a laboratory scale for research purposes. From a pharmaceutical industry perspective, spray-drying and hot-melt extrusion are the most common methods of preparation, as reflected in the preparation methods reported for the marketed amorphous drug formulations (163,164). The broad use of a spray-drying technique may be due to the fact that it is relatively easy to scale this method to industrial settings, and to apply it to a wider range of physicochemical properties, especially for compounds with thermal and shear instability. In addition, the method is material-sparing compared to other methods and this attracts formulation scientists to use laboratory-scale spray drying in the early development phase when the amount of material available may be limited (165–167).

**Fig. 4:**
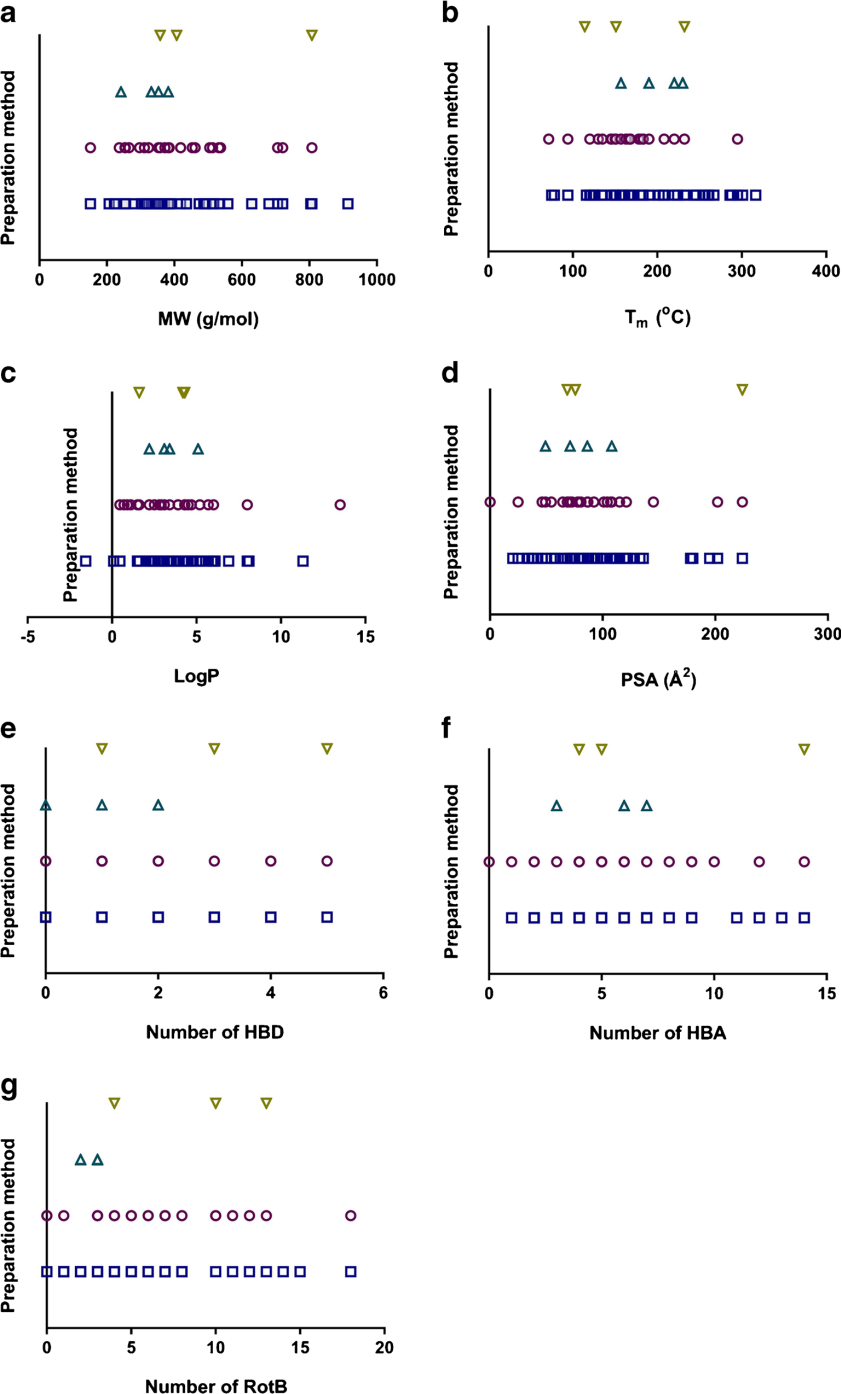
Relationship between the physicochemical properties of the model compounds and the chosen amorphous formulation preparation method: solvent-based (), temperature-based (), mechanical-based () and combination (). *MW* molecular weight, *T*_*m*_ melting point, *Log P* partition coefficient between octanol and water, *PSA* polar surface area, *HBD* number of hydrogen bond donors, *HBA* number of hydrogen bond acceptors, *RotB* number of rotatable bonds.

Beside reviewing different amorphization methods, we also wanted to investigate whether there is any relationship between selection of the preparation method and the physicochemical properties of the compounds. The results of this analysis are summarized in Fig. 4. A trend was observed with the temperature-based and solvent-based methods, but not with mechanical-based and combined preparation methods, probably because of the limited number of studies where these methods were used. The choice of solvent-based methods was made across a slightly wider distribution of MW, T_m_ and logP than seen with temperature-based methods. This was based on analysis of the single properties (Fig. 4) as well as the principal component analysis (data not shown). It does make sense that the solvent-based methods, which typically use water and/or organic solvents with variable dielectric constants ranging from relatively polar (e.g. methanol) to non-polar (e.g. hexane or mixtures of solvents), have the capacity to handle compounds with a wide range of lipophilicity. The trend for the temperature-based methods to be somewhat less applicable to the typical brick-dust molecules (T_m_ > 200°C) is probably the result of the increased risk of chemical degradation when using the required high temperature. For other physicochemical properties (number of rotatable bonds, hydrogen bond capacity, molecular complexity) there was no clear trend for predicting the method.

**Fig. 5:**
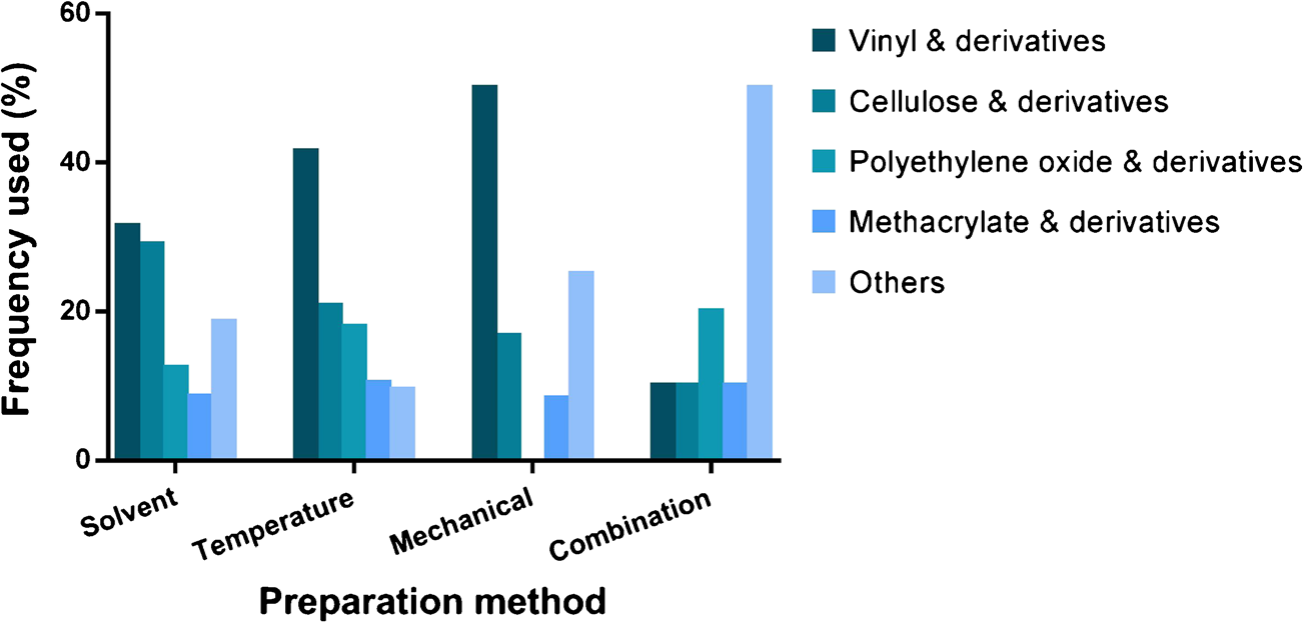
Distribution of excipients used in the different methods of preparing amorphous formulations.

Finally, we investigated the extent to which different excipients were used in the various preparation methods, focusing on polymers commonly used in amorphous formulations. Because the role of the excipients in the reviewed amorphous formulations was not limited to stabilizing the amorphous form, and because the roles were not specified in the reviewed articles, this was difficult to analyze (Fig. 5) (71–85,87,88,90–139,141–161,166,168–177). The polymers were most commonly (62%) used in solvent-based methods. This finding parallels our earlier observation that there is wide usage of solvent-based methods in the preparation of amorphous formulations. Vinyl (31%) and cellulose (29%) polymer derivatives were the most common choice for solvent-based methods, followed by polyethylene oxide derivatives (12%) and methacrylate polymers (9%). For the temperature-based methods, the vinyl derivatives were the most commonly used (42%), followed by cellulose derivatives (21%), polyethylene oxide derivatives (18%), and methacrylate polymers (10%). Mechanical-based methods commonly used vinyl polymers (50%), followed by cellulose (17%) and methacrylate (8%) polymers. The mild conditions associated with solvent-based methods (i.e. relatively lower temperature and mechanical activity) compared to temperature-based and mechanical-based methods make them more flexible in terms of selecting the best compound and excipient, as long as both components are miscible and soluble in the solvent system used. However, more stringent criteria are required for temperature-based methods, including criteria for the T_g_ and the T_m_ of both the compound and the excipient, the viscosity of the excipient, the miscibility of the components, the extrudability of the mixture, and the potential degradation of the compound on exposure to high temperature during the process (74,76,103,109,129,138,150,155,175). These could limit the applicability and use of temperature-based methods in the preparation and study of amorphous formulations.

Recently, reports on a novel processing technology based on temperature and high shear force mixing have shown potential in overcoming the limitations associated with conventional hot-melt extrusion and spray-drying techniques (143,147,151). More uncommon preparation methods which are not widely used and established industrially for the preparation of amorphous formulations such as supercritical fluid impregnation /solvent-antisolvent (84,92,102,149), electrospinning /electrospraying (72,91,141,153,174), confined impinging jet (107) and pressurized gyration (161) have also been explored even though the applicability in large scale is still a question.

### Methods of Assessing the Performance of Amorphous Formulation

Amorphous material is thermodynamically unstable, with an inherent tendency to return to its more stable crystalline form. In order to ensure that developing an amorphous drug formulation is a viable strategy for improving solubility, the stability of the formulation in the solid state (physical) and in solution (supersaturation) must be examined. If crystallization occurs during storage, the solubility and dissolution advantages obtained from the amorphous formulation will be lost. In the reviewed articles, despite the pharmacopoeia and international harmonization initiative providing guideline methods for stability studies for any dosage form, the conditions and durations selected for the physical stability studies of amorphous formulations were surprisingly diverse. The temperatures ranged from 2°C to 60°C and the relative humidity varied between 0% and 100%. The duration also varied significantly, with stability studies lasting from 24 h to two years (73,74,81,82,84,86,88,92,93,104,105,109,110,113,115,116,118,120,123,124,126,127,129,130,132,134,135,139,143,146,149,153,158,166,169,172,175,176). While most of the studies did not mention the container used for the physical stability test, a few specified, for example, whether a closed or open container was used (88,92,113,123,132,146). Of these six articles that mentioned whether closed or open container was used, only one specifically reported the type of container used (i.e. high density polyethylene bottle) in the stability study (132) whereas the remaining studies mentioned only that tube (88), airtight brown vial (92), vial (113), sealed aluminum strip (123) or sealed glass container (146) was used. These large variations in the design of stability studies make direct comparison impossible, since the stability profile obtained is specific only for the stability method employed. Hence, what was regarded as a stable amorphous formulation in one study may not be stable in another. While the stability tests in the reviewed articles may have been designed to answer a particular research question, it would of course be beneficial for the research field if these studies also were performed using more standardized methods (USP) and/or follow the universally harmonized guidelines. Information could then be extracted about the molecular processes involved in the loss of the (solid) amorphous form.

**Fig. 6:**
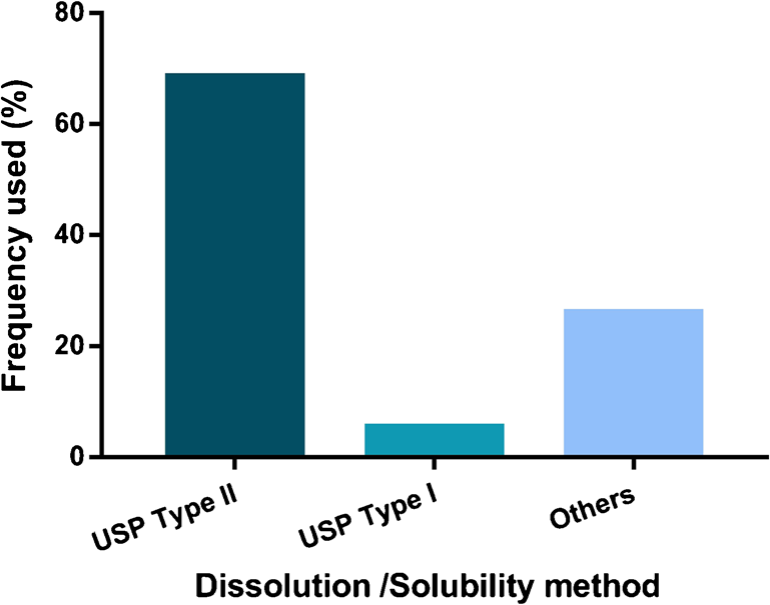
*In vitro* dissolution apparatuses used in the reviewed studies.

Solubility and *in vitro* dissolution studies are also conducted to assess the performance of an amorphous formulation. In the reviewed articles, no specific method was used in the investigation of amorphous solubility and dissolution behavior, even though different pharmacopoeia methods provide general guidance on how they should be performed. Ninety-two of the 101 studies reported solubility and dissolution studies (71–74,77–79,81–92,95,97–107,109–111,113–119,121,123,124,126–133,135–139,141–154,156,157,160,161,166,168–176). The USP *in vitro* dissolution type II apparatus was the most commonly used instrument (67%), followed by the USP type I apparatus (6%), while the remaining 27% used various other apparatuses, including modified versions of the USP type I only (148) or paired with confocal Raman microscopy (98), USP types I and II apparatus (72), USP type IV (152), closed loop of USP types II and IV (152), a perspex flow cell (73,142), a rotary mixer (131), a Chinese pharmacopoeia type III apparatus (118), an in-house miniaturized USP type II apparatus (175), Sirius T3 apparatus (146), μFLUX dissolution-permeation apparatus (141), Raman UV-Vis flow cell system (99), a centrifuge (88), high throughput screening using a 96-well plate (82,115,157), Wood’s apparatus (99,137), an orbital shaking incubator (156), and another shake-flask method (171) (Fig. 6). In several of these studies, dissolution apparatuses were either modified, optimized or paired with other instruments and methods to enable the investigation, monitoring, and understanding of other processes that take place concurrently with dissolution such as permeation (141), release mechanisms (73,98,99,101,106,142) and wetting kinetics (71). The experimental conditions during *in vitro* dissolution testing (e.g. type of medium, medium volume, temperature, pH, and sink/non-sink conditions) also varied. The majority of the studies used a large volume of medium (≥250 ml), which can indirectly be translated to the equally large amounts of materials required in practice. Depending on the condition selected (sink or non-sink), weight of samples between 5–1800 mg were reported (72,74,77–79,83–85,87,90,91,95,100,105,106,109–111,114,116,121,126,128,130,135,136,138,149,154,166,170). Interestingly, the use of small-scale or miniaturized *in vitro* dissolution methods is still limited. Likewise, the media reported were mainly different types of a simple buffers such as treated water (i.e. deionized, degassed, distilled, purified), HCl, and phosphate/acetate buffers at pHs ranging from 1.2 to 7.4 (71–74,77–79,81–88,90–92,95,100–107,109–111,113–119,121,126,127,129–133,135–139,149,150,153,154,156,160,161,166,168–177). Among the 88 studies that reported a dissolution assay, only sixteen (~18%) used a biorelevant dissolution medium (BDM) such as simulated gastrointestinal fluids and simulated saliva (77,79,82,97,121,123,130,135,136,145,152,154,160,169,170,176). These disparities in medium used affect the conclusions, which (as with the stability studies) make head-to-head comparison difficult. The choice of solubility and dissolution methods was possibly partly driven by the general goals of the studies performed.

## The Need for Methods to Better Understand the Processes Occurring during the Preparation, Storage and Dissolution of Amorphous Drugs

**Fig. 7:**
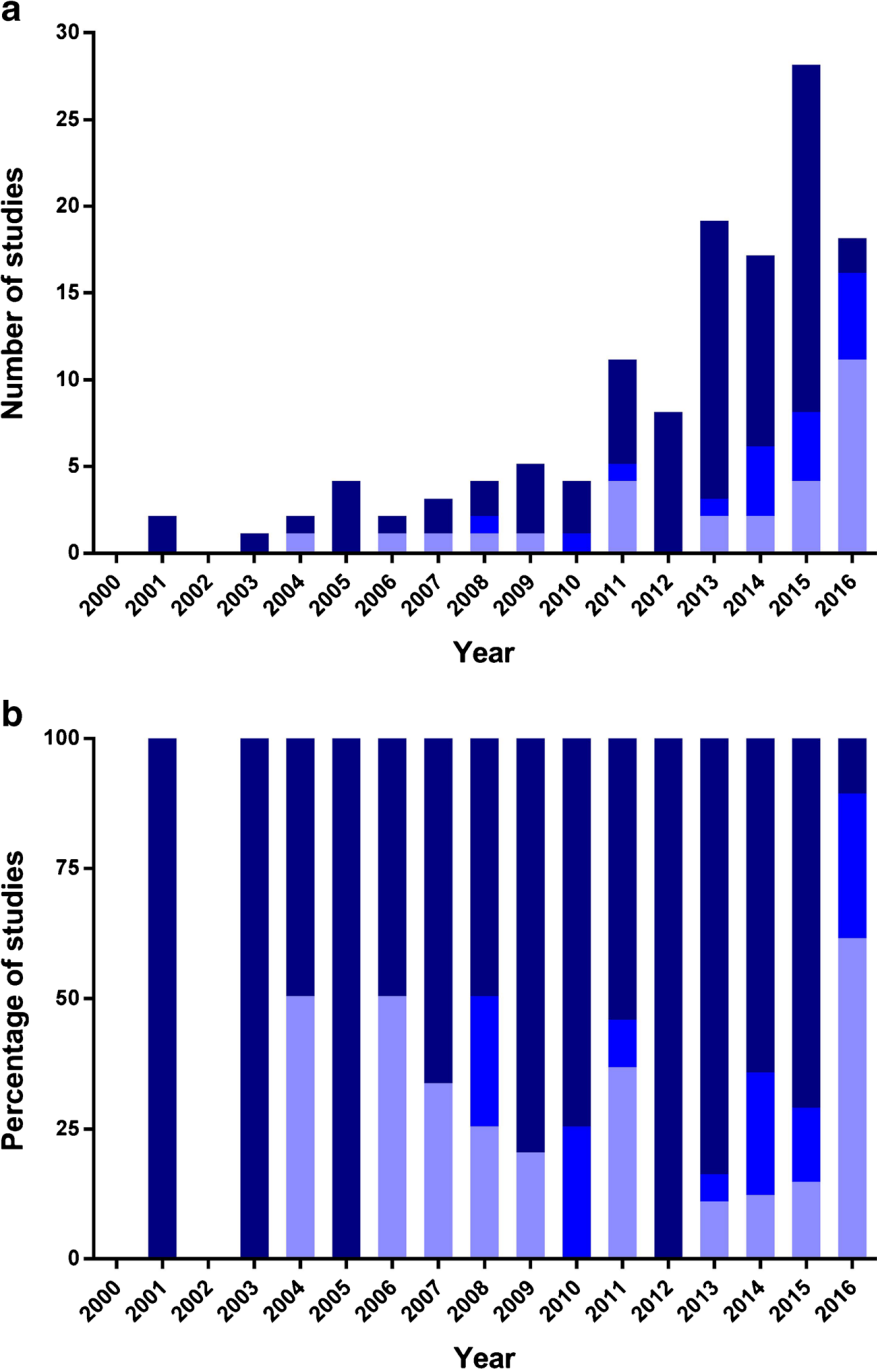
The (**a**) number and (**b**) percentage of amorphous drug-based studies reported between 2000 and 2016 that focused on research (), a combination of research and development (), or development ().

Our analysis of articles on amorphous compounds published 2011–2016 indicates that knowledge of when to use specific methods or excipients to produce a well-functioning delivery system based on the amorphous form is still limited. One reason for this limited knowledge is simply the low number of compounds that have been studied. Typically, each study investigated only one or at best a few compounds and the general applicability of the conclusions drawn cannot therefore be validated. More than 65% of the studies focused on the development stage rather than the research stage of the R&D process and seemed to be aiming to quickly assess the development potential of an amorphous form of a particular compound (Fig. 7). Encouraging is however that there has been a tremendous increase in research-based studies reported in 2016 which are included in this review (61%), an indicator that there is an increased awareness within the scientific community on the better understanding of amorphous-based formulation which performance is influenced by complex interplay between multiple factors. We would like to emphasize the importance of increasing the focus on the research stage so as to increase understanding of when amorphization should be targeted for a new compound. A scientific rationale needs to be developed to guide the choice of methodology and excipients for any new compound (rather than just starting to explore a number of excipients and methods).

In a recent study by Edueng *et al*., an experiment-based road map for delineating the need for stabilization of the amorphous form dissolved in water was developed (177). The experimental set up was designed to show whether the compound had solid-to-solid or solution-mediated crystallization, with the aim of clarifying whether stabilizers should be included in the solid amorphous material or whether a simple physical mixture with, for example, polymers would be enough to maintain supersaturation when the material was exposed to water. The method was based on a number of solid-state and dissolution characterization methods, all of which used small amounts of compound to measure the response. A solvent-based method (spray-drying) was used to prepare the material, but other methods could potentially have been used to produce the amorphous form. However, based on our analysis of method selection in this paper, which indicates that the solvent-based method seems to be more generally applicable from a compound perspective, it makes sense to start with any of the solvent-based method such as spray-drying, solvent evaporation or freeze drying when aiming for an amorphous formulation. In the study by Edueng and coworkers, spray-drying was the method of choice due to its established applicability in the production of amorphous compounds and/or ASD both in laboratory and industrial scale (177). The experimental road-map is one way of increasing the throughput of compounds being explored. The process allows larger datasets to be studied and these are expected to provide a scientific basis for better understanding the mechanisms of, for example, crystallization and stabilization of the amorphous material. Further, if structurally diverse datasets are explored, it will be possible to extract general information about these processes, which would help to guide the development of future amorphous formulations of any new compound that is in need of increased solubility or dissolution in order to be orally administered.

### Assessment of glass-forming ability: new experimental and computational methods

GFA and crystallization tendency have recently been explored computationally using a number of models based on multivariate data analysis (as described in the “selection of model compound” section). The large datasets studied and multivariate data analysis such as partial least square analysis and support vector machines have allowed discrimination between glass formers and non-glass formers, and even between compounds with rapid or slow crystallization rates in the solid form, based on calculated molecular descriptors obtained from a 2D or 3D chemical structure (60,63–65). Models which can predict the T_g_ have also been described recently (178). These models are useful in the early stages of formulation design for indicating whether amorphization of a new compound is likely to be successful and the extent to which stabilization of the solid form might be required.

Recently, Blaabjerg and coworkers suggested the use of time-temperature-transformation (TTT) diagrams for identifying the GFA (179). While all compounds are theoretically able to transform into their amorphous form given optimal experimental settings, in this method the GFA is based on the cooling rate that is needed to produce the amorphous form. Based on experimental data for 12 compounds they suggested that compounds that are extremely difficult to make amorphous (and which in the computational work mentioned above would be defined as non-glass formers) require cooling rates >750°C/min. Glass formers were then divided into those requiring modest cooling rates (>10°C/min; the rate typically seen in standard melt-quenching methods) and those requiring low cooling rates (>2°/min); these compounds should be easily transformed to the amorphous form and hence are ideal compounds for this formulation pathway (179). An efficient work flow for this pathway would be to identify glass formers using a computational model and then to verify the prediction using the TTT method.

### Some New Insights to the Phase Behavior of ASDs

The possibility to experimentally assess drug-polymer miscibility has been dependent on the development of new high-resolution techniques for detection of small phase domains in amorphous solids. Beside the scanning probe based imaging techniques, (180) NMR has been proven as a powerful tool for identifying domains in phase separated amorphous solids down to a few nanometer in size (162,181). The relationship between thermodynamic miscibility, phase separation and crystallization of ASDs are however not yet fully clarified.

On the other hand, new insights to the phase behavior of drugs in supersaturated solutions generated during dissolution of ASDs have recently been provided by Taylor and co-workers. The formation of sub-micron particles of felodipine and indomethacin was observed during dissolution of ASDs which distorted UV-probe measurements leading to the detection of falsely high free drug concentrations (182). In later publications liquid-liquid phase separation was proven to occur upon precipitation, which occurs at concentrations exceeding the solubility of the amorphous form of a drug (168). This underpins that a thorough understanding of the phase behavior during dissolution of these types of systems is crucial for rational selection of formulation and assessment techniques for ASDs.

### Computational Simulations of Amorphous Drugs, Amorphous Solid Dispersions, and Supersaturation

While the experimental road-map established by Edueng *et al*. reveals the effect of water as a plasticizer of the amorphous form and the extent to which supersaturated aqueous systems are prone to nucleate and crystallize, advanced computational models can also be used to study amorphous compounds and their formulations (177). During recent years, molecular dynamics (MD) simulations have been used to study the GFA, the characteristics of the amorphous form itself, the interaction between the compound and water, and the possible crystallization of the compound from supersaturated solutions. MD simulation is a powerful computational technique that can reveal inter- and intra-molecular interactions at a detailed level. Depending on the resolution of the simulation, the length-scale and the physical volume that can be explored may vary; the most computer-demanding all-atom simulations are typically used to study simulation boxes of ~10 nm (each side) for 100 ns, whereas the less resolved, coarse-grained method is used for boxes of ~50 nm (each side) for microseconds. Both of these methods have their place; however, most studies so far have used the all-atom resolution. It should be mentioned that although this methodology is beginning to be applied in studies of amorphous drug systems, it is still only used in relatively few studies and is not used to the same extent as in material sciences and cell biology studies. Some of these studies are outlined in the following section to provide a glimpse of the level of information that can be expected from these simulations.

#### Molecular Dynamics (MD) Simulations of Amorphous Drugs, Amorphous Solid Dispersions, and Crystallization from Supersaturation

MD simulations have been used by Xiang and Anderson to study the characteristics of amorphous indomethacin with and without stabilizing polymers (89,183). They also performed MD simulations for polymers such as hypromellose acetate succinate and poly-lactide (184,185). The investigations used dry amorphous indomethacin, as the simulation only used 0.6% *w*/w water molecules (183). The system was quickly equilibrated to a high temperature (10 ns at 600 K and 1 bar under periodic boundary conditions) after which it was cooled to 200 K using a cooling rate of 0.03 Kps. This procedure mimicked the setup for melt quenching; however, the rate of cooling was much higher in the simulations than what is possible to achieve experimentally. As a consequence, the T_g_ in the simulations deviated significantly from those determined experimentally (64 K higher). However, dynamic properties such as density, water diffusion in the material, and the rotational relaxation of indomethacin were in good agreement with the experimental data. It was also found that the hydrogen bond pattern in amorphous indomethacin was highly complex, which is in agreement with spectroscopic data of amorphous indomethacin. In the subsequent study they investigated the interaction between indomethacin and PVP, using the simulations to aid, for example, investigation of the miscibility of the drug and the excipient. The importance of the interactions between the compound and the polymer were also analyzed, and it was found that PVP stabilized the indomethacin via formation of hydrogen bonds (89). Fule and Amin also used MD simulations to explore the stabilizing effect of polymers in the solid form (186). They studied the interaction between posaconazole and a number of different excipients, using the monomer form of the polymers investigated. For each of the drug-polymer systems, the strength of the bonds between the drug and the polymer was used to indicate the stabilizing effect of the polymers. The most stable system in the MD simulations was also the highest ranked formulation in *in vitro* and *in vivo* dissolution tests, although the experimental data did not provide a statistical analysis.

Mesoscale dissipative particle dynamic (DPD) simulations can be used to computationally explore the movement of long polymer chains over relatively long time spans (187,188). This method was recently used to study the performance of a 20% lacidipine (BCS II drug)-loaded ASD stabilized by Eudragit E 100 when exposed to pH 1.2 and pH 6.8 dissolution tests (189). A large number of experimental techniques were used to characterize the ASD and the data analysis was complemented with a DPD simulation to investigate the experimental observations at a molecular level. The DPD simulation facilitated insight into the miscibility of the included components and the effects of pH on the miscibility of, for example, the drug and water or the polymer and water. It was also demonstrated that swelling of the polymer was important for increasing the release of lacidipine. Overall, the DPD simulation allowed the successful study of the microscale properties (i.e. miscibility, swelling, drug release) although the molecular interaction pattern was not revealed at the low resolution used. For that purpose other simulations with increased resolution, e.g. at the all- or united-atom level, are needed.

Nucleation and crystal growth from a supersaturated solution have also been studied using MD simulations (190). Investigation of the impact of the supersaturation level on the second nucleation of bicalutamide (a BCS class II drug), i.e. nucleation after introducing a bicalutamide crystal to seed production of a particular polymorph, revealed that the second crystallization occurred in the supersaturated solvent at high supersaturation levels. This is to be expected, as the chemical potential of the system would have driven homogeneous crystallization to occur. In contrast, crystallization occurred on the surface of the seed crystal when the supersaturation level was low. This too is expected, since the concentration was too low to cause the significant aggregation needed to form the crystal nuclei in the solution. The crystallization occurring at intermediate supersaturation levels was a mixture of these two processes. Solubilized aggregates of the drug were formed in the intermediate supersaturated solution and these crystallized when they came into contact with the seed crystal. The “second” crystal was then able to detach from the crystal seed surface because of the weak bonding forces and small areas of contact between the flat seed crystal and the curved aggregate that crystallized. Once the crystals had detached they could induce crystallization of the solubilized aggregates when these collided in the solution, acting as second seed crystals in the solution.

## Summary

The field of amorphous drug formulations needs to move from being descriptive science to becoming predictive science. It is clear from this review that most of the recent publications on amorphous dosage form design and selection of excipients were still focusing on development, although the papers published during 2016 were indeed more research focused than those published 2011–2015. It may be acceptable for industrial-based studies to focus on the development stage, but academic institutions need to set the science first. To develop the field further, researchers need to ask (and try to answer) the difficult, but crucial, questions related to the mechanisms and processes involved in the performance of the formulation. The research-based and development-based practices within these different sectors (academic and industry) should be complementary in nature, in order not to unnecessarily replicate studies already done, thus accelerating the whole developmental process. The recent advances in technology, including highly sensitive experimental techniques (based on spectroscopy, microscopy, scattering, or thermal techniques) and computational methods (quantitative structure property relationships obtained via multivariate data analyses of varying complexity and MD simulations), should be used together to tackle processes that we still poorly understand. If these processes were better understood, the probability of developing a well-functioning ASD at the first attempt might be significantly increased. Some of the crucial processes that merit further attention and significant effort to increase the understanding of these complex formulations are outlined below. It is suggested that successful research in this field would consequently be rapidly translated into the development setting.

- Processes occurring at the particle surface during storage (at interfaces exposed to varying temperatures and humidity) and during dissolution (at the particle-water interface) merit further attention. In particular, we need studies of:
  - diffusion of water through the amorphous form and the effect of water on relaxation and nucleation kinetics,
  - the effect of elevated temperature and its relationship to the kinetics of nucleation,
  - the likelihood of a highly viscous interface forming between a solid amorphous material and water during dissolution and the potential effect of this on release and nucleation kinetics,
  - nucleation occurring in the highly concentrated microclimate surrounding the amorphous particle during dissolution.
- Insights into the molecular interactions between the drug and the polymer in the solid material, as well as during dissolution, are crucial in order to arrive at a scientific rationale as to why a certain polymer is successful. These studies require large numbers of drugs and polymers and varying conditions, and would benefit from an experimental design approach where combination effects can be more easily identified. Further, the obtained data could be used to produce models of a more general applicability. The key to success is to take a global approach, where head-to-head comparisons can be performed between different drugs and polymer systems. Hence, the field needs to move away from case studies, which often explore either reference (model) compounds or polymers that have been repeatedly used in the scientific literature.
- Dissolution/release and nucleation/crystal growth processes under physiologically relevant conditions need attention to understand the interplay between the compound and the excipients of the ASD and the naturally present (soluble) lipoidal nanoaggregates. This area needs standardization to make use of data generated by different laboratories. Further, dissolution experiments performed in combination with permeability assessments, as suggested by, for example, Yamashita and colleagues, would reveal the extent to which the release of drug from ASDs is directly related to increased absorption (and, hence, could be expected to increase the bioavailability), and should also increase understanding of the *in vivo* processes driving absorption (191).

These processes are already being addressed in some laboratories. However, we are emphasizing the need for a more holistic approach, in which a combination of experimental data, theory, and computational simulations are the cornerstones of each research project, in an effort to extend the borders of research on amorphous pharmaceutical materials. The field needs larger databases on the properties of amorphous compounds and the associated formulations, which include data that have been determined by sensitive but standardized methods. In addition, scientists need to challenge themselves, and the field, by asking the truly difficult questions related to amorphous formulations, moving from the comfort zone we are currently in. If this can be achieved, we will start to reveal the mysteries around the nucleation process that takes place when ASDs are exposed to water, the interplay between the water, the drug, and the excipient, and the supersaturation levels and absorption achieved *in vivo*.

## Abbreviations

API: Active pharmaceutical ingredient
ASD: Amorphous solid dispersion
BCS: Biopharmaceutics classification system
BDM: Biorelevent dissolution medium
DPD: Dissipative particle dynamics
DSC: Differential scanning calorimetry
GFA: Glass forming ability
HBA: Number of hydrogen bond acceptors
HBD: Number of hydrogen bond donors
HBN_eff_: Effective hydrogen bond number
HCl: Hydrochloric acid
logP: Octanol-water partition coefficient
MD: Molecular dynamics
MW: Molecular weight
NMR: Nuclear magnetic resonance
PSA: Polar surface area
PVP: Polyvinylpyrrolidone
R&D: Research and development
Ro5: Lipinski’s rule-of-five
RotB: Number of rotatable bonds
T_g_: Glass transition temperature
T_m_: Melting point
TTT: Time-temperature-transformation
USP: United States pharmacopoeia
